# Trib2 expression in granulocyte-monocyte progenitors drives a highly drug resistant acute myeloid leukaemia linked to elevated Bcl2

**DOI:** 10.18632/oncotarget.24525

**Published:** 2018-02-19

**Authors:** Caitriona O’Connor, Krishna Yalla, Mara Salomé, Hothri Ananyambica Moka, Eduardo Gómez Castañeda, Patrick A. Eyers, Karen Keeshan

**Affiliations:** ^1^ Paul O’Gorman Leukaemia Research Centre, Institute of Cancer Sciences, College of Medical, Veterinary and Life Sciences, University of Glasgow, Glasgow, G12 0XB, UK; ^2^ Department of Biochemistry, Institute of Integrative Biology, University of Liverpool, Liverpool L69 7ZB, UK

**Keywords:** Trib2, AML, chemotherapy, BCL2

## Abstract

Trib2 pseudokinase has oncogenic and tumour suppressive functions depending on the cellular context. We investigated the ability of Trib2 to transform different haemopoietic stem and progenitor cells (HSPCs). Our study identified the granulocyte-macrophage progenitor (GMP) subpopulation as a potent leukaemia initiating cell of Trib2-driven AML *in vivo*. Trib2 transformed GMPs generated a fully penetrant and short latency AML. AML cells expressing elevated Trib2 led to a chemoresistant phenotype following chemotherapy treatment. We show that Trib2 overexpression results in an increase in BCL2 expression, and high Trib2 expressing cells are highly sensitive to cell killing by BCL2 inhibition (ABT199). Combined treatment with chemotherapeutic agents and BCL2 inhibition resulted in synergistic killing of Trib2+ AML cells. Trib2 transformed GMP AML cells showed more chemoresistance compared with HSPC derived Trib2 AML cells associated with higher *Bcl2* expression. There is significant correlation of high *TRIB2* and *BCL2* expression in patient derived human AML cells. These data demonstrate that the cell of origin influences the leukaemic profile and chemotherapeutic response of Trib2+ AML. Combined TRIB2 and BCL2 expression in AML cells may have clinical utility relevant for monitoring drug resistance and disease relapse.

## INTRODUCTION

Tribbles homolog 2 (TRIB2) pseudokinase functions as both an oncogene and a tumour suppressor with important roles in human health and disease. TRIB2 is involved in the aetiology of a number of cancers including acute leukaemia, melanoma, ovarian, lung and liver cancer [1]. While kinase activity has not yet been linked to the tribbles family of proteins (TRIB1, TRIB2 and TRIB3), they function via protein scaffolding to regulate signalling pathways including AKT and MAPK signalling, and to control protein levels via the proteasome including key cell differentiation and cell cycle regulators [2].

TRIB2 is thought to be the earliest evolved family member of the tribbles family [2]. It has been shown to have oncogenic and tumour suppressor activities in cancer cells, which appear to be dependent on the cellular context [1]. As an oncogene, Trib2 is capable of driving acute myeloid leukaemia (AML) that is dependent on the proteasomal degradation of the transcription factor C/EBPα [3]. This function was also key to the oncogenic role of TRIB2 in lung cancer, and was shown to be mediated via scaffolding with the ubiquitin E3 ligases COP1 and TRIM21 [4, 5]. The modulation of AKT phosphorylation and expression of the cell cycle regulator CDC25C are also associated with the oncogenic activity of TRIB2 [6, 7]. In contrast, TRIB2 has tumour suppressor activities in acute leukaemia, linked with aberrant MAPK signalling that resulted in tumour cell death [8–10].

TRIB2 expression has been reported as a biomarker for diagnosis and progression of cancers including melanoma [11]. For leukaemia, TRIB1, TRIB2 and TRIB3 expression associates with a number of the different subgroups of AML; TRIB1 is both mutated and elevated in acute megakaryocytic leukaemia (AMKL) and AML with 8q amplifications [12–14]; TRIB2 expression associates positively with the AML subset with dysregulated *CEBPA* [15, 16] and negatively with subgroups with a high frequency of mutated *NPM1* and *FLT3,* however TRIB2 is high in AML with *FLT3TKD* [10, 17]. TRIB3 expression associates positively with APL disease progression [18, 19]. TRIB2 (and TRIB1) expression promotes AML in HOX-dependent leukaemias [16, 20–22], and in acute promyelocytic leukaemia (APL) [23]. Thus, TRIB proteins represent promising leukaemia biomarkers and therapeutic targets in AML.

AML cells comprise a heterogeneous population arranged in a hierarchical manner with a small number of leukaemia stem cells (LSCs) responsible for initiating and maintaining the disease, and which are the cause of drug resistance and disease relapse [24]. It is hypothesised that the cell of origin or leukaemia initiating cell (LIC) may influence disease progression, LSC phenotype and response to therapy. Comparing AML LSCs with their normal counterparts, studies have indicated that LSC activity occurs in cells not only within the hematopoietic stem cell (HSC) compartment but also in multipotential progenitors (MPPs) or more committed myeloid progenitors (common myeloid progenitors (CMPs), granulocyte-macrophage progenitors (GMPs)). In human AML, LSC activity can be found not just in the CD34^+^CD38^-^ cell, but also present with the lowest frequency in the more mature CD38^+^ fraction [25–28]. However, cellular heterogeneity exists within the LSC fraction, and LSCs also exhibit plasticity. Thus characterising the LSC population does not identify the original cell that gave rise to the leukaemia. It has been clearly demonstrated that cells other than HSCs acquire LSC properties when transformed by appropriate oncogenes, which are characterized by their ability to transfer self-renewal potential to cells at committed stages of differentiation [29–34]. When ectopically expressed in hematopoietic stem and progenitor cell (HSPC) enriched bone marrow (BM) cells, Trib2 was shown to induce AML in a murine transplant model [3]. It remains unclear whether the LIC in a Trib2+ leukaemia is a HSC or a more committed progenitor cell.

Our current study identified the LIC in Trib2+ AML. Expression of Trib2 in the GMP cell drove a highly penetrant disease with a short latency in a murine transplant model. To address whether the chemotherapeutic response is affected by Trib2 expression and the nature of the LIC, we assessed the response of Trib2+ AML cells to chemotherapeutic agents used commonly in AML treatment. We provide evidence for a TRIB2 role in chemoresistance via elevation of BCL2 expression and reveal synergistic cell killing following co-treatment with BCL2 inhibition and standard chemotherapeutic drugs. Our results highlight that using TRIB2 as a biomarker, we can identify AML cells with a heightened sensitivity to combined BCL2 inhibition and chemotherapy, thus providing a novel therapeutic approach for treating TRIB2+ AML.

## RESULTS

### Trib2 can transform HSCs, MPPs, CMPs, GMPs and MEPs *in vitro*

**Figure 1:**
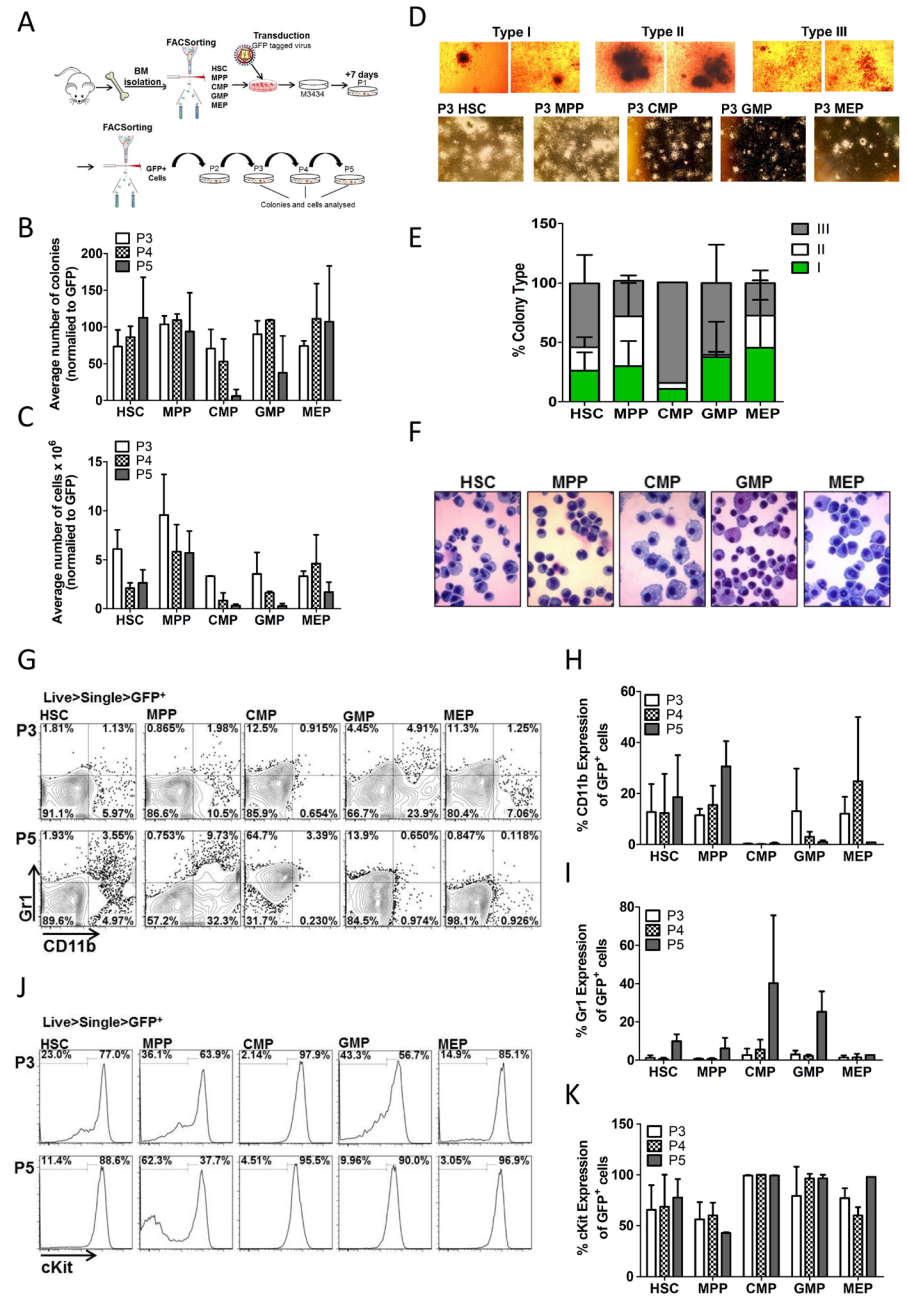
Trib2 transforms all HSPC populations with varying efficiency and immunophenotypes **(A)** Schematic overview of CFC experiments. Murine BM cells were isolated and sorted for HSC, MPP, CMP, GMP and MEP populations. Following transduction the Trib2 expressing cells were plated in M3434 methylcellulose media 24hrs later. After 7 days (P1) colonies had formed, cells were sorted for GFP expression and GFP^+^ cells were serially replated to P5. Colonies and cells were analysed at P3, P4 and P5. **(B)** Average number of colonies at each replating normalised to GFP expression. Averages are of 2 independent experiments each with 3 technical replicates shown +/− SD. **(C)** Average number of cells produced at each replating. Averages are of pooled triplicate plates from 2 independent experiments +/− SD. **(D)** Representative images of colony types at P3 under 4x magnification (top) and 2x magnification (bottom). **(E)** Distribution of colony types at P5. Bars represent the average of 2 independent experiments each with 3 technical replicates +/− SD. **(F)** Cellular morphology of Trib2 transformed stem and progenitor cells. Cells from P3 colonies were spun onto glass slides using a cytocentrifuge and stained with the KwikDiff staining system. Representative images are shown, taken under 40X magnification. **(G)** Cells from each replating were stained for CD11b and Gr1 and analysed by flow cytometry. Representative flow cytometric analysis of GFP^+^ Trib2transformed stem and progenitor cells from P3 and P5. Graphs represent the average percentage expression of CD11b **(H)** and Gr1 **(I)** of GFP^+^ Trib2transformed stem and progenitor cells from P3, P4 and P5. Data represent the average of 2 independent experiments +/− SD. **(J)** Representative flow cytometric analysis of GFP^+^ Trib2 transformed stem and progenitor cells from P3 and P5 for cKit expression. **(K)** Graphs represent the average percentage expression of cKit on GFP^+^ Trib2 transformed stem and progenitor cells from P3, P4 and P5. Data represents the average of 2 independent experiments +/− SD.

To identify the cell of origin in murine Trib2+ leukaemias, an *in vitro* approach was first utilised. Several studies have explored the LIC of specific oncogenes using either a methylcellulose-based serial replating assay and/or retroviral-mediated murine bone marrow transplantation (BMT) [29–34]. Purified stem and progenitor cells, including HSCs, MPPs, CMPs, GMPs and megakaryocyte-erythroid progenitors (MEPs), transduced with a GFP-tagged lentiviral vector encoding *Trib2*, were seeded in methocult supplemented with cytokines that induce myeloid differentiation (Figure 1A). Following the first plating round (P1), GFP^+^ cells were sorted and serially replated. The ability of cells to form colonies by the third replating in this assay implies acquisition of self-renewal ability and increased proliferation. The cells were serially replated to P5, and colonies and cells were analysed from P3 to P5. Control transduced cells are unable to replate [4]. All Trib2 transduced populations were efficiently producing colonies at P3, and these cells still formed colonies by P5, however different efficiencies between groups were observed (Figure 1B). By P5, CMP derived cells were producing very few smaller sized colonies (Figure 1C). Trib2 transduced GMP derived cells also produced smaller sized colonies, while MPP derived cells produced the largest sized colonies. Colonies were also categorised based on their morphology as described previously [34] and were of 3 distinct phenotypes: Type I, Type II and Type III. Type I colonies contain immature myeloid precursor cells and compact in size, Type II colonies contain more mature cells and have a compact centre surrounded by a diffuse halo of cells, while type III colonies are the most differentiated and comprise dispersed cells with no core. Immature Type I colonies were well represented on transformed HSC, MPP, GMP and MEP plates, comprising 25-50% of colonies by P3 but much fewer on CMP plates (Figure 1D and 1E). The intermediate Type II colonies were rarely observed in Trib2 transduced CMPs or GMPs, being most prominent on transformed MPP plates, as well as HSC and MEP plates. Interestingly, while type III (mature) colonies were present on all plates, they were predominantly on CMP and GMP plates with CMP plates being primarily composed of type III colonies. Cytospins of the cells demonstrated that Trib2 transduced HSC, MPP, GMP and MEP derived colonies contained many undifferentiated cells with a high nucleus/cytoplasm ratio, while CMP derived colonies contained very few undifferentiated cells, with almost all cells showing evidence of granulocytic or monocytic differentiation (Figure 1F).

It was next determined if the differences in colony size and shape, and cellular morphology correlated with cell surface marker changes. Flow cytometric analysis of P3 and P5 cells revealed that HSCs, MPPs and MEPs had similar CD11b and Gr1 expression, both the CMP and GMP derived colonies trended toward lower CD11b and higher Gr1 (Figure 1G-1I). CD11b and Gr1 expression increased slightly in HSC and MPP derived cells as the cells were replated to P5, likely because the time taken to mature from these hierarchical stem populations. There was high cKit expression, a marker of cell immaturity, in almost all populations at each plating, although less than 50% of MPP derived cells expressed cKit by P5 (Figure 1J and 1K). Analysis of CD11b and Gr1 expression was performed through the cKit^+^ and cKit^-^ gates which showed no CD11b expression and low Gr1 expression on cKit^+^ cells, while as expected the cKit^-^ cells expressed mature myeloid markers (Supplementary Figure 2A and 2B). The high levels of cKit expression observed in HSC, CMP, GMP and MEP derived Trib2 transduced cells correlates well with published data showing Trib2-induced AML cells are cKit^+^ [3]. Together these data show that Trib2 can transform stem and progenitor populations *in vitro* with different efficiencies, and the resultant transformed colonies and cell types differ depending on the cell of origin. The data shows that Trib2 has conferred both self-renewal ability and a differentiation block to HSCs, GMPs and MEPs. While Trib2 transduced MPPs also displayed self-renewal ability, by P5 there was a large population of cKit^-^ cells still present which express the myeloid differentiation markers CD11b and Gr1. These data indicate that the Trib2 LIC potentially resides in either the HSC or GMP as only these populations display pre-leukaemic characteristics *in vitro*.

### Trib2 propagates a fully penetrant and short latency AML from the GMP *in vivo*, but not the HSC, MPP or CMP

**Figure 2:**
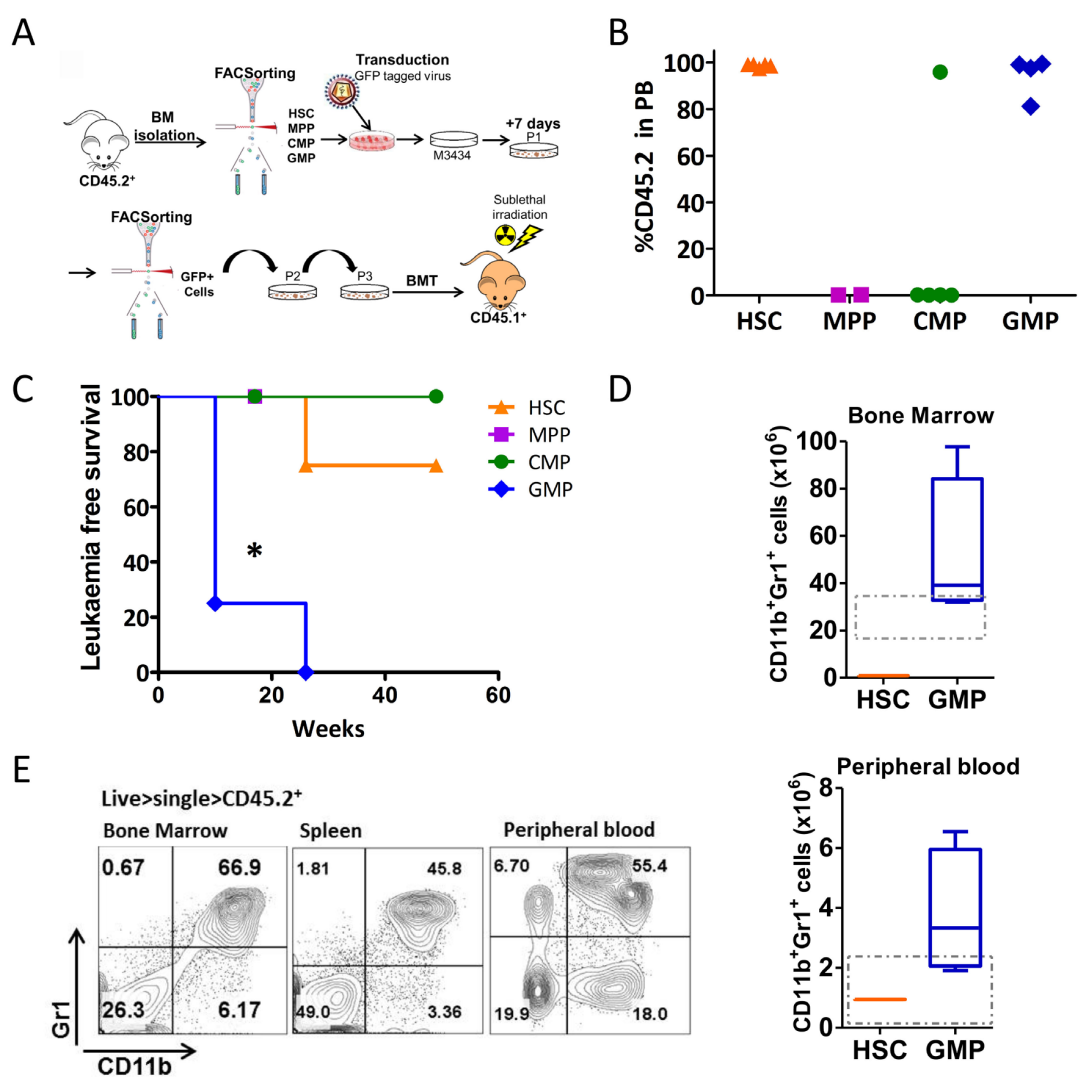
Trib2 transformed GMPs drive AML with full penetrance and short latency **(A)** Schematic overview of transplantation model used to identify the Trib2 LIC. CD45.2^+^ sorted HSCs, MPPs, CMPs, and GMPs were transduced with Trib2 lentivirus and plated in M3434 methylcellulose media after 24hrs. After replating to P3, cells were transplanted into sub-lethally irradiated CD45.1^+^ recipient mice. **(B)** Peripheral bleeds were taken at 7 weeks post BMT and analysed by flow cytometry for CD45.2 donor cell expression. Each dot represents percentage CD45.2 expression in the PB of an individual mouse, HSC (n=4), MPP (n=2), CMP (n=5), GMP (n=4). **(C)** Kaplan-Meier survival curve of mice reconstituted with cells derived from Trib2 transduced HSCs (n=4), MPPs (n=2), CMPs (n=5) and GMPs (n=4). ^*^P=0.0171 by Log-rank test. **(D)** Absolute numbers of CD11b^+^Gr1^+^ cells in the BM (top) and PB (bottom) of Trib2 transduced HSC transplanted (n=1) and GMP transplanted (n=4) leukemic mice. Box and whiskers plot with whiskers denoting min to max, and line representing mean values. Dashed box denotes normal range in healthy mice as determined by HemaVet 950FS reference intervals (PB) and previously published data (BM). **(E)** Representative flow cytometric analysis of mature myeloid (CD11b, Gr1), in the BM, spleen and PB of Trib2 transformed GMPs (L-GMPs). Data is representative of n=4 GMP mice.

The results from the serial replating assay indicated that Trib2 conferred replating and self-renewal capacity to the HSC, MPP, CMP, and GMP populations. Despite this, Trib2 was unable to efficiently block differentiation in the MPP and to fully transform the CMP derived cells. In order to resolve these observations and to elucidate the leukaemia initiating potential of the stem and progenitor populations, a BMT was performed. By P3 of the serial replating assay all populations were exhibiting hallmarks of pre-leukaemic cells i.e. efficiently producing colonies, high cKit expression and low expression of mature myeloid markers. Thus the CD45.2^+^ cells from P3 HSC, MPP, CMP and GMP derived colonies were transplanted into sublethally irradiated CD45.1^+^ C57BL/6 recipient mice and transplanted animals were monitored for 1 year (Figure 2A).

Peripheral bleeds (PB) were taken at 7 weeks post-transplant and the levels of engraftment were determined by quantifying CD45.2 expression in the blood (Figure 2B). Cells transplanted from HSC and GMP derived colonies displayed high levels of engraftment. Cells transplanted from MPP-derived colonies showed no engraftment in the recipient mice, while only 1 of 5 CMP transplanted mice displayed engraftment in the PB at 7 and 16 weeks post-transplant. The MPP and CMP transplanted mice that displayed no engraftment in the PB were culled at 16 weeks post BMT and all had normal white blood cell (WBC) counts, normal spleen weights, and normal percentages of circulating neutrophils, lymphocytes, monocytes, eosinophils and basophils (Supplementary Figure 3A-3C). All mice transplanted with GMP derived cells succumbed to disease with a median latency of 14 weeks (Figure 2C and Table 1). This is significantly shorter than AML disease developed from HSPC (5FU or cKit enriched) BM cells previously reported which had a median latency of 26 weeks [3]. Of the cohort transplanted with HSC derived cells, only 1 of 4 mice developed disease within 1 year, with a latency of 26 weeks. Further analysis of the mice that displayed peripheral engraftment but did not develop leukaemia within 1 year, showed no disruption to haemopoiesis with normal spleen weights, WBC counts and normal percentages of circulating neutrophils, lymphocytes, monocytes, eosinophils and basophils (Supplementary Figure 3D-3F). The leukaemias generated from Trib2 transduced GMP derived cells were all AMLs, identified as CD11b^+^Gr1^+^ cells (Figure 2D and 2E) and a lack of expression of B and T cell markers. Analysis of the absolute cell numbers in the BM and PB of mice with disease demonstrated that mice transplanted with Trib2 transduced GMPs had elevated myeloid blast cells compared to the HSC transplanted mouse (Figure 2E). The sole HSC derived disease had a longer latency and a phenotype that was different from that of the GMP. BM cells of the HSC transplanted mouse had some CD11b expression with little to no Gr1 expression, and almost all cells expressing the B cell marker B220, but not CD19, and a fraction of cells expressing the T cell marker CD8 but not CD4 (Figure 2D and data not shown). These data show that Trib2 cannot induce leukaemia from MPPs or CMPs *in vivo*. Leukaemia is generated from the HSC and GMP *in vivo*, with different efficiencies, and has the ability to generate different types of leukaemia depending on the cell of origin. A uniformly myeloid disease with 100% penetrance was generated from the Trib2 transduced GMPs (leukaemic GMP or L-GMP) identifying the GMP as a potent Trib2 LIC.

**Table 1:** Summary table of the outcome of the *in vivo* leukaemogenesis experiment, specifying the cells of origin (column 1), donor cells (column 2), disease outcome (column 3), disease latency (time to death column 4) and mean time to disease in the last column

Immunophenotype of the transduced cells	Input BM	Phenotype of the Leukemia arising	Time to death (Weeks)	Mean time to AML +/− SD (weeks)
Lin-cKit+Sca1-CD34+CD16/32hi	GMP MiGR1 TRIB2	Myeloid	10	14 +/− 8 weeks
Lin-cKit+Sca1-CD34+CD16/32hi	GMP MiGR1 TRIB2	Myeloid	10	
Lin-cKit+Sca1-CD34+CD16/32hi	GMP MiGR1 TRIB2	Myeloid	10	
Lin-cKit+Sca1-CD34+CD16/32hi	GMP MiGR1 TRIB2	Myeloid	26	
Lin-cKit+Sca1-CD34+CD16/32+	CMP MiGR1 TRIB2	No disease	16	N/A
Lin-cKit+Sca1-CD34+CD16/32+	CMP MiGR1 TRIB2	No disease	16	
Lin-cKit+Sca1-CD34+CD16/32+	CMP MiGR1 TRIB2	No disease	16	
Lin-cKit+Sca1-CD34+CD16/32+	CMP MiGR1 TRIB2	No disease	16	
Lin-cKit+Sca1-CD34+CD16/32+	CMP MiGR1 TRIB2	No disease	49	
Lin-cKit+Sca1+CD150-CD48-	MPP MiGR1 TRIB2	No disease	16	N/A
Lin-cKit+Sca1+CD150-CD48-	MPP MiGR1 TRIB2	No disease	16	
Lin-cKit+Sca1+CD150+CD48-	HSC MiGR1 TRIB2	Mixed lineage	26	26 weeks
Lin-cKit+Sca1+CD150+CD48-	HSC MiGR1 TRIB2	No disease	49	N/A
Lin-cKit+Sca1+CD150+CD48-	HSC MiGR1 TRIB2	No disease	49	
Lin-cKit+Sca1+CD150+CD48-	HSC MiGR1 TRIB2	No disease	49	

### Trib2 expression mediates sensitivity to AML chemotherapy

**Figure 3:**
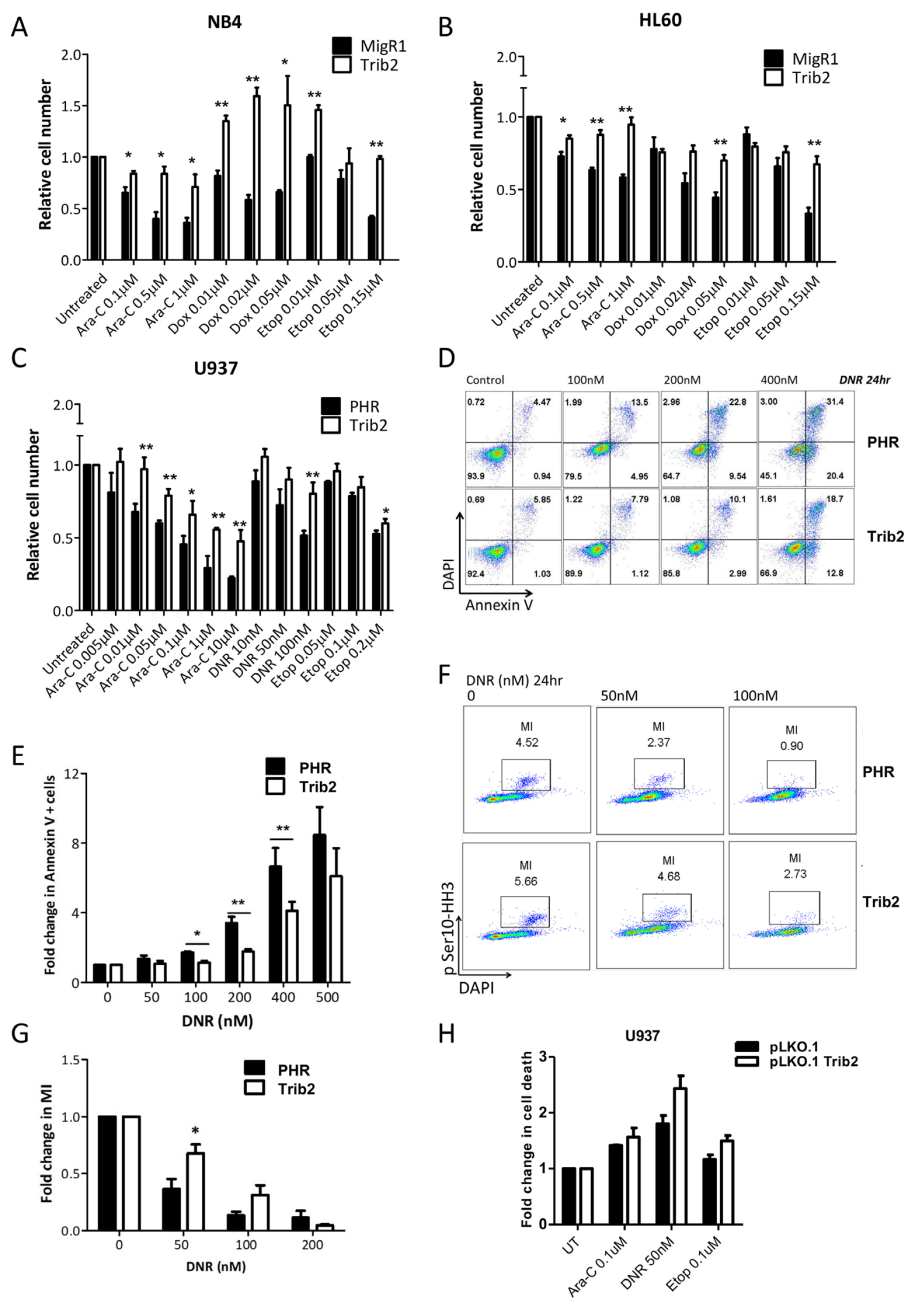
Trib2 overexpressing cells are chemotherapy resistant Cell viability of **(A)** NB4 **(B)** HL60 **(C)** U937 overexpressing Trib2 cells, in response to increasing doses of cytarabine (Ara-C), Doxocyline (Dox), daunorubicin (DNR) and Etoposide (Etop) after 24hr, determined by Trypan blue exclusion. **(D)** Representative apoptosis profile of DNR treated U937 cells transduced with Trib2 or PHR empty vector for 24hr; live, DAPI−Annexin V−; early apoptotic, DAPI− Annexin V+; late apoptotic, DAPI+ Annexin V+. **(E)** Graph of the average fold change in cells positive for Annexin V, in U937 cells transduced with Trib2 or PHR empty vector and treated with DNR for 24hr, from 3 independent experiments. Error bars denote +/− SEM. **(F)** Representative mitosis FACS profile of DNR treated U937 cells transduced with Trib2 or PHR empty vector for 24hrs. **(G)** Graph of fold change in mitotic index (MI), following treatment with DNR for 24hr and normalized to untreated control sample. Error bars denote +/− SEM. **(H)** U937 cells were transduced with control pLKO.1 or pLKO.1 shTrib2 lentivirus and treated with cytarabine (Ara-C), daunorubicin (DNR) and etoposide (Etop). Fold change in cell death (Annexin+ and/or DAPI+ cells) was determined after 24hrs. Bars represent the average of 2 independent experiments each with 3 technical replicates. Error bars denote +/− SEM.

The LSC is thought to be the cell that evades chemotherapy and drives relapse. To study the response of cells expressing elevated Trib2 to AML chemotherapeutic drugs, we used NB4, HL60 and U937 human AML cell lines. Cells were transduced with control empty vector (MigR1 retrovirus or PHR lentivirus) or with MigR1 Trib2 or PHR Trib2, followed by treatment with increasing concentrations of cytarabine (Ara-C), doxorubicin (DOX) or daunorubicin (DNR) (both DOX and DNR are anthracyclines that can be used interchangeably), and etoposide (Etop). Analysis of cell viability showed that Trib2 overexpressing cells were significantly more resistant to the chemotherapeutic agents compared to control cells (Figure 3A-3C). Assessment of apoptosis using Annexin V/DAPI staining confirmed that elevated Trib2 expression reduced early (Annexin V+/DAPI-) and late apoptosis (Annexin V+/DAPI+) following drug treatment (Figure 3D and 3E). To address if cell death was being evaded as a result of an effect of Trib2 on cell proliferation, we measured the number of cells that remained in mitosis followed drug treatment. Drug treated Trib2 expressing cells were able to evade apoptosis and continue to undergo mitosis compared to control treated cells (Figure 3F and 3G). As previously published, loss of Trib2 in AML cells results in cell growth inhibition and G1 cell cycle arrest [35]. Still, with lentiviral mediated loss of Trib2 expression in U937 cells, we observed increased cell death in response to drug treatment when normalised to untreated cells (Figure 3H). Together these data show that human AML cells expressing high levels of Trib2 are resistant to chemotherapeutic drug treatments.

### Trib2 expression increases BCL2 expression which contributes to Trib2-mediated chemoresistance

**Figure 4:**
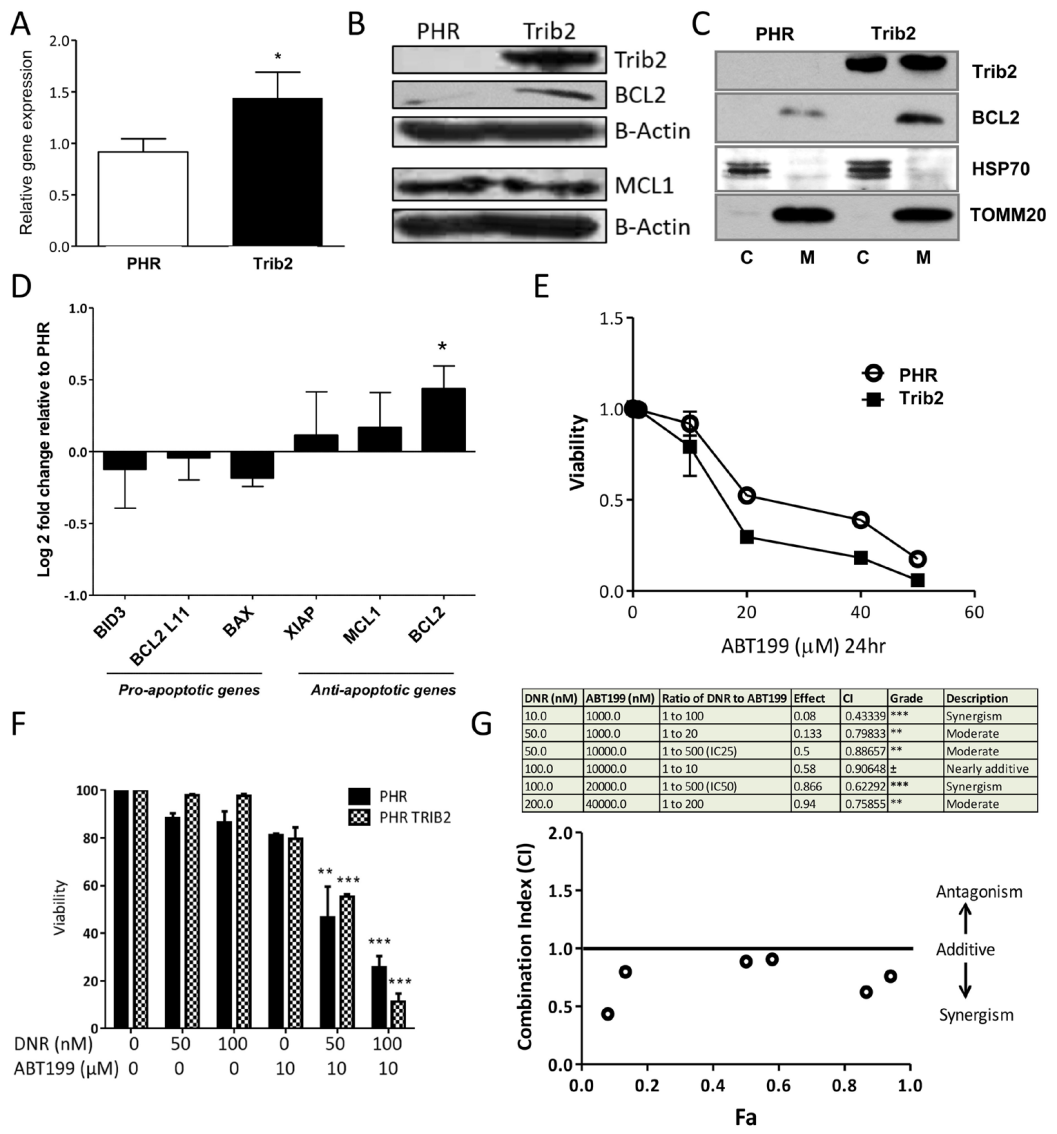
Trib2 overexpression increases BCL2 expression and sensitises to BCL2 inhibition-induced cell death **(A)** Graph represents fold change (2^-ddCt^) of *BCL2* gene expression in Trib2 over expressing U937 cells relative to empty vector control. ABL was used as the house keeping gene. Bars represent average of n=3. Error bars denote +/− SEM. For statistical analysis an unpaired, two-tailed, student *t*-test was performed. **(B)** Western blot analysis of whole cell lysates from U937 expressing PHR Trib2 or control PHR cells for Trib2, BCL2, MCL1 and β-ACTIN (loading control) expression. Representative blot of 2 biological replicates. **(C)** Western blotting analysis of fractions enriched for Mitochondria (M) and cytoplasmic (C) from U937 expressing PHR Trib2 or control PHR cells for BCL2 and Trib2. HSP70 and TOMM20 used as controls for cytoplasm and mitochondrial separation. Representative blot of 3 biological replicates. **(D)** Real time PCR was performed in U937 expressing PHR Trib2 or PHR control cells for the indicated genes. Gene expression was normalised to housekeeping gene ABL. Gene expression in PHR Trib2 cells was plotted relative to PHR cells, represented by –ddCt or Log_2_ fold change. Data represents the average of 3 independent experiments. **(E)** Cell viability of U937 expressing PHR Trib2 or control PHR cells in response to increasing doses of ABT199 after 24hr, determined by Annexin V and DAPI stained cells. Cells Annexin V- and DAPI- were plotted as normalized percentage of cells to untreated control. An average of 3 biological replicates was plotted as an xy scatter plot as a dose response curve. Error bars denote +/− SEM. **(F)** Bar chart presentation of cell viability of PHR and PHR Trib2 expressing U937 cells in response to the indicated concentrations of DNR and ABT199 determined by FACS based analysis of Annexin V and DAPI stained cells. Cells Annexin V- and DAPI- were plotted as normalized percentage of cells to untreated control. Average of 2 independent experiments graphed with error bars denoting +/− SEM, and statistical significance determined by student *t* test. **(G)** Table depicting various drug combination treatments of U937 expressing PHR Trib2 cells, ratio of the drug concentrations (DNR:ABT199), fraction effected (represented by cells undergoing apoptosis or cells Annexin V+, determined by FACS), combination index values generated by CompuSyn software and the extent of synergism as indicated by the numerical value of Combination Index (CI). Data is representative of 3 independent experiments. CI values were plotted against Fa (fraction affected).

Drug resistance as a result of continued proliferation and an inability to undergo apoptosis has been linked to levels of the BCL2 family of apoptotic regulators [36]. We therefore measured the expression levels of BCL2 in Trib2 overexpressing cells. *BCL2* gene and protein levels were highly upregulated in human AML U937 cells transduced with Trib2 compared to control cells (Figure 4A and 4B). Anti-apoptotic BCL2 proteins reside on the outer mitochondrial membrane and prevent apoptosis by inhibiting the activation of the pro-apoptotic family members BAX and BAK. We confirmed that Trib2 leads to the elevation of mitochondrial BCL2 levels (Figure 4C) conferring an anti-apoptotic phenotype on Trib2 expressing cells. In addition to elevated BCL2, Trib2 overexpression lead to trending increases in anti-apoptotic and pro-survival expressing genes *MCL1* and *XIAP* (Figure 4D), although protein levels of MCL1 were not affected (Figure 4B). We also observed trending decreases in pro-apoptotic genes *BID3*, *BAX* and *BCL2L11* but these were not significant (Figure 4D). To directly assess whether the increase in BCL2 expression by Trib2 contributed to the resistance to chemotherapy, we first assessed the sensitivity of control and Trib2 overexpressing cells to the BCL2 inhibitor ABT199, a BH3 mimetic [36]. Cells overexpressing Trib2 were more sensitive to death induced by BCL2 inhibition alone compared to control cells (Figure 4E). Next, we determined the ability of BCL2 inhibition to sensitize Trib2 expressing AML cells to chemotherapy. Combination treatment of ABT199 and DNR significantly reduced the survival of Trib2 overexpressing human AML cells compared to DNR treatment or BCL2 inhibition alone and compared to control cells (Figure 4F). We used the Chou-Talalay method to calculate the combination index (CI), whereby the drugs are added in a given ratio and the CIs calculated using CompuSyn software to give an indication of synergism, where CI<1 is synergism, CI=1 is additive, and CI>1 is antagonism. These results revealed significant synergism between BCL2 inhibition and DNR treatment in Trib2 expressing human AML cells (Figure 4G).

### High TRIB2 and BCL2 expression correlate and contribute to a drug resistance phenotype

**Figure 5:**
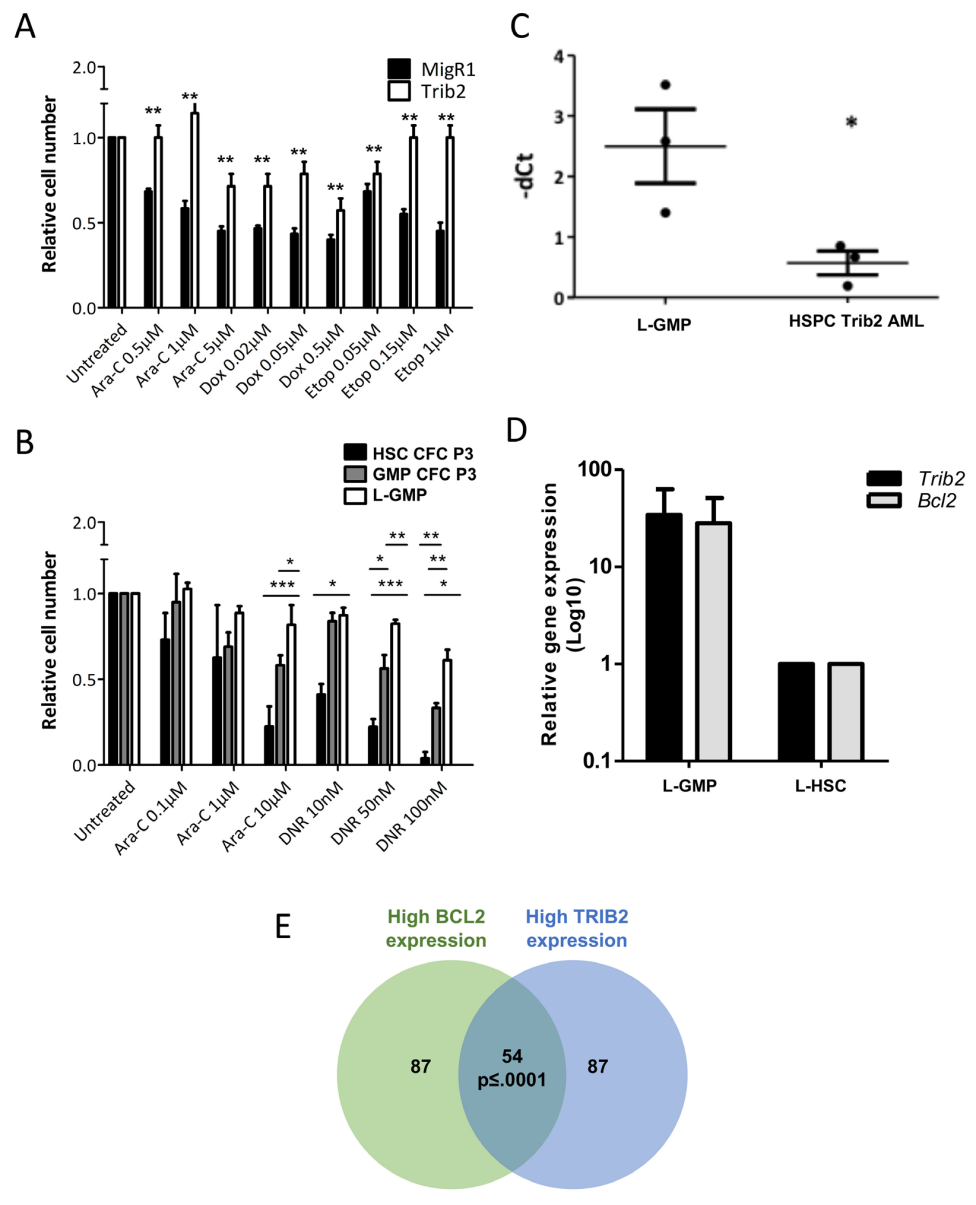
Trib2 L-GMP is highly drug resistant with correlation of high TRIB2 and BCL2 expression Cell viability of AML cells derived from Trib2 transduced HSPCs **(A)** and Trib2 transduced HSC and GMP CFC3 cells, and AML cells (L-GMPs) derived from Trib2 transduced GMPs **(B)**, following treatment with the indicated concentrations of Ara-C and DNR and assessed for live cells at 24hours by trypan blue exclusion. **(C)** Transcript expression of Bcl2 in Trib2 L-GMPs and HSPC Trib2 AML cells determined by real time PCR. HPRT1 housekeeping gene was used to normalise the gene expression. Each data point represents an individual mouse in the respective arm. P<0.05 determined by student *t* test. Error bars represent +/− SEM. **(D)** Gene expression analysis of *Trib2* and *Bcl2* in Trib2 L-GMP (n=4) and L-HSC (n=1) cells determined by real time PCR. HPRT1 housekeeping gene was used to normalise gene expression. Bars represent average of group and error bars represent +/− SEM. **(E)** Venn diagram showing a strong expression correlation between *TRIB2* and *BCL2* expression, generated by filtering AML samples from the MILE study. p≤ 0.0001, empirically assessed after 10,000 Monte Carlo simulations.

To determine whether AML generated *in vivo* from Trib2 overexpression similarly led to a chemoresistant profile, we studied the effect of drug treatment (Ara-C, Dox/DNR and etoposide) on AML cell viability. Murine AML cells derived from Trib2 expression in cKit enriched haemopoietic stem and progenitor cells (HSPCs, which contain HSCs, MPPs, CMPs and GMPs) were significantly more resistant to the chemotherapeutic drugs than control transduced cells (Figure 5A). We then asked whether the Trib2 L-GMP that was capable of driving a fully penetrant and short latency AML *in vivo*, exhibited drug resistance. In comparison to the *in vitro* transformed HSCs and GMPs, *in vivo* derived AML cells from Trib2 transformed GMPs displayed significant resistance to AML chemotherapeutic drugs (Figure 5B). We have then correlated high *Bcl2* expression with drug resistance in the Trib2 L-GMP. High levels of *Bcl2* expression were observed in Trib2 L-GMP cells compared to Trib2 AML cells generated from an HSPC enriched population (Figure 5C). When we compared *Bcl2* expression in Trib2 L-GMP cells with cells from the single HSC derived disease (L-HSC), we also observed higher *Bcl2* expression in the L-GMPs and this correlated with higher *Trib2* expression (Figure 5D). We then correlated high *BCL2* expression and high *TRIB2* expression in human AML by interrogation of a publically available human AML dataset [37]. Analysis of samples within the top 25 percentile of *TRIB2* and *BCL2* expression showed a significant correlation of *TRIB2* and *BCL2* expression (Figure 5E). These data indicate that the GMP population is readily transformed by Trib2 and is highly drug resistant correlating with high *BCL2* expression. Together, these data indicate the GMP as the potential LSC in AMLs expressing high levels of Trib2. Monitoring patients for a high *TRIB2/BCL2* expressing cell may identify patients that may benefit from combination BCL2 inhibition and chemotherapy, and provide further information on patients at risk for chemotherapy resistance and disease relapse.

## DISCUSSION

Our study has identified the GMP as a potent LIC for the pseudokinase Trib2. Trib2 transformed GMPs produced a fully penetrant myeloid leukaemia with fast kinetics and an immunophenotype consistent with a maturing myeloid cell (L-GMP). We report that the Trib2 L-GMP is a highly drug resistant cell, evading cell death induction by commonly used AML chemotherapeutic drugs. Due to the increase in BCL2 expression, mediated by high Trib2 expression, Trib2+ AML cells can be sensitized to standard chemotherapies by combined treatment with BCL2 inhibition. In 2016, the US Food and Drug Administration (FDA) approved the use of the BCL2 inhibitor ABT199 (Venetoclax, Venclexta®) for the treatment of patients with CLL with 17p deletion. In July 2017, the US FDA granted breakthrough therapy designation for Venclexta® in combination with low dose cytarabine (LDAC) for elderly patients with previously untreated AML who are ineligible for intensive chemotherapy. We have provided evidence allowing for the identification of AML cells that would be targeted by this treatment and potentially patients that would benefit from this clinical strategy.

Trib2 overexpression was able to confer self-renewal and proliferative capacity on stem and progenitor populations *in vitro* but the cell of origin affected the number, size and morphology of colonies and cells produced. However, *in vivo* Trib2 was only able to initiate leukaemia with potency from the GMP, with incomplete penetrance from the HSC, and not at all from the CMP and MPP. The median latency of the GMP derived disease was just 14 weeks while the latency of the HSC derived disease was significantly longer at 26 weeks. It is possible that the GMP disease may have enriched for the Trib2 LIC compared to the slower HSC derived disease, although if this was true then a more penetrant disease should have been observed with the HSC derived cells. Previous studies support that the LIC can be enriched in the HSC [25, 27, 30] or a more mature myeloid progenitor population such as the GMP [28, 33, 38–40] and that the identity of the LIC can affect the disease latency. A study of primary AML samples showed that LSC activity was enriched in subsets which phenotypically resembled murine LMPPs and GMPs [28]. When examined at the molecular level the leukaemic LMPPs and GMPs resembled their normal counterparts suggesting that the LIC was a committed progenitor cell as opposed to a HSC with aberrant surface marker expression.

Our data suggest that distinct mechanisms of Trib2 leukaemogenesis exists dependent on the cell of origin being a HSC or GMP. Endogenous *Trib2* expression is higher in the HSC compared to the GMP (Supplementary Figure 1A and 1B), and in our overexpression model we achieved high Trib2 expression in the HSC and the GMP following transduction (Supplementary Figure 1D). The highly penetrant disease generated by Trib2 expression in the GMP may be attributable to the expression levels of lineage specific genes. C/EBPα is essential for the CMP to GMP transition and its expression is highest in the GMP (Supplementary Figure 1C), and as Trib2 degrades C/EBPα to drive AML disease [3, 4], this may account for the potent transformability of the GMP. Indeed, our recent work has also shown that in the absence of C/EBPα, Trib2 in unable to generate AML disease [41]. Recent studies have put forward an interesting hypothesis that myeloid differentiation is required for the formation of LSCs and for AML initiation [42]. The authors showed that leukaemia could be restored in *Cebpa* deficient cells by treating cells with myeloid cytokines, thus demonstrating that it was not loss of C/EBPα itself, but rather impaired myelomonocytic differentiation which prevented leukaemia initiation. Therefore, the differentiation state of the cell of origin may determine the leukaemic potential of cells with elevated Trib2 expression and our data would support this. However, the ability of Trib2, and other oncogenes, to generate leukaemia from functionally and phenotypically distinct stem and progenitor populations also suggests that there are overlapping gene expression signatures initiated by different oncogenes which facilitate leukaemic transformation [31]. Gene expression analysis has revealed many shared pathways between HSCs and LSCs [43], thus suggesting that oncogenes switch on stem cell transcriptional programs. Purification of the LSC compartment in an MLL-AF9 mouse model has indicated that at the molecular and phenotypic level the MLL-AF9 LSC closely resembled a more committed progenitor cell, the GMP which also expressed HSC specific genes [30]. However, the phenotype of LSCs in established leukaemias may be determined by the oncogenic event rather than reflecting the phenotype of the originating cell, and thus the phenotype of LSCs may not be indicative of the cell of origin. Indeed using a conditional inducible genetic model, it has been shown that disease developed by MLL-AF9 expression in a HSC results in a more aggressive phenotype compared to expression in the GMP [44]. Other studies have isolated HSCs and HSPCs and retrovirally introduced oncogenes in these cells to identify the cells of origin. Specific fusion oncoproteins found in human AML patients MLL-ENL, MLL-AF9, MLL-GAS7, BCR-ABL and MOZ-TIF2 can transform HSCs, CMPs and/or GMPs [29, 32, 45]. In summary, studies support our conclusion that the GMP, with its inherent differentiation state and gene expression signature, is a more potent LSC following Trib2 expression compared to other progenitors.

We have identified that Trib2 leads to increased BCL2 expression, an anti-apoptotic and pro-survival gene that is therapeutically targetable, and associated with drug resistance across multiple cancers [46]. The expression of apoptotic proteins in AML is highly heterogeneous, and intrinsic to the disease pathology. High levels of *BCL2* have been shown to be associated with a poorer survival and treatment resistance [47]. The potential of BCL2 as a therapeutic target is already well recognised. Present day clinical trials are largely focusing on the second generation, selective BH3 mimetic ABT199, as the early generation inhibitors have been limited by thrombocytopenia [48]. As a single agent only a small number of AMLs are susceptible, thus combination therapies are being pre-clinically and clinically assessed e.g. combination with LDAC, kinase inhibitors, hypomethylating agents, Mcl1 and mdm2 inhibitors [49] (e.g. NCT02203773, NCT02427451). The sensitivity to the BCL2 inhibitor ABT199 positively correlates with endogenous BCL2 protein level and negatively correlates with BCL-XL protein level in AML cell lines.

Our work has shown that BCL2 expression is upregulated by Trib2 and contributes to a chemoresistant phenotype highlighting that combination treatment with chemotherapy and BCL2 inhibition would overcome this drug resistance. Previously it has been shown that overexpression of Trib2 in Me-1 cell line led to dephosphorylation of BCL2 associated with an apoptotic response [10], and that BCL2 overexpression could rescue the apoptosis induced by Trib2 overexpression in TF-1 cell line [50], however total endogenous BCL2 levels or other BCL2 family members were not reported. In contrast, BCL2 overexpression could rescue apoptosis induced by Trib2 knockdown in T-ALL cell lines [51], which is in line with our previous data that Trib2 knockdown in AML cells also induced apoptosis [35] and with our current findings. The difference in the response of AML cells to TRIB2 overexpression between the former studies and ours may be explained by the balance of pro- and anti-apoptotic BCL2 family members endogenous expression in the AML cell lines used. We have shown in our study that Trib2 induced expression of BCL2 but does not affect MCL1 expression. One possible mechanism for the induction of BCL2 expression by Trib2 is through C/EBPα and NF-κB. C/EBPα p42 and p30 have been shown to cooperate with NF-κB p50 to induce Bcl-2 transcription, and *CEBPA* and *Bcl-2* RNA levels correlate highly in human AMLs [52]. While Trib2 overexpression degrades C/EBPα p42 to induce AML, it also results in an increase in the truncated p30 isoform of C/EBPα, and this increase may contribute to the increase in BCL2 expression observed. Considering the fact that endogenous levels of C/EBPα are much higher in the GMP than the HSC, this may also account for the comparatively low levels of BCL2 observed in the L-HSC cells.

The oncogenic activity of TRIB2 has been linked to its role in the block of myeloid differentiation, degradation of key myeloid transcription factors and cell cycle regulators, and pro-survival mechanisms. In contrast, studies have revealed tumour suppressor functions of Trib2, including pro-apoptotic and reduced MAPK signalling. These opposing functions have been described in acute leukaemia. Elevated levels of Trib2 drive a potent AML disease in murine models and associate with a specific subgroup of human AML, both human and mouse models are linked to the C/EBP transcription factor family. The knockdown of TRIB2 leads to cell cycle arrest and apoptosis of the cancer cells [3, 35, 51, 53]. In contrast, low levels of *TRIB2* associate with human AML with high NPM1 and FLT3ITD mutation frequency and in some cell types, elevated TRIB2 drives apoptosis [10, 50]. High levels of TRIB2 have been associated with drug resistance to standard therapies and PI3K inhibitors in a number of solid cancers [6], whereas low levels of TRIB2 expression have been associated with drug resistance in ovarian cancer patients [54]. These conflicting roles of TRIB2 may be due to the genetics of the AML subtype, the cancer type, and the profile of the apoptotic regulators within those cells.

In summary, elevated Trib2 expression conferred a drug resistant phenotype in AML which we have correlated with high *Bcl2/BCL2* expression. Combined therapy using BCL2 inhibitors and standard chemotherapy has potential to eradicate a clone of human AML cells that exhibits drug resistance, identifiable by high expression of BCL2 and TRIB2.

## MATERIALS AND METHODS

### Cell culture and fluorescence activated cell sorting (FACS)

NB4, HL-60 and U937 cell lines were maintained in RPMI plus 10% FBS, 1% Penicillin/Streptomycin/Glutamine. Peripheral blood was isolated by tail vein nick or by cardiac puncture using 1ml insulin syringe. Long bones were removed and bone marrow (BM) cells were isolated by crushing in a pestle and mortar. In all tissues red blood cells (RBC) were lysed in RBC lysis buffer (155mM NH4Cl, 10mM KHCO3, 0.1mM EDTA). When isolating primary stem and progenitor populations, total BM cells were first MACS (Miltenyi) purified by lineage depletion (CD3, CD4, CD8, B220, CD11b, Gr1, Ter119). Cells were incubated and sorted with the appropriate antibodies; HSCs (Lin^-^cKit^+^Sca-1^+^CD150^+^CD48^-^), MPPs (Lin^-^cKit^+^Sca-1^+^CD150^-^CD48^-^), CMPs (Lin^-^cKit^+^Sca-1^-^CD34^+^CD16/32^+^), GMPs (Lin^-^cKit^+^Sca-1^-^CD34^+^CD16/32^hi^), and MEPs (Lin^-^cKit^+^CD34^-^CD16/32^-^). Sorting was performed using a BD FACS Aria II (BD Biosciences).

### Trib2 transduction

PHR and PHR Trib2 lentivirus (SFFV promoter) or MigR1 and MigR1 Trib2 (MSCV promoter) retrovirus, as previously described and used interchangeably [35, 41] were used to overexpress Trib2 in murine primary cells and human AML U937, HL60 and NB4 cell lines. pLKO.1 and pLKO.1 Trib2 lentivirus, as previously described [35], were used to knockdown Trib2 in human AML U937 cells. HEK293T packaging cell line were calcium phosphate transfected with retroviral or lentiviral expression plasmids, together with retroviral (pCGP, VSV-G) or lentiviral (psPax2, VSV-G) packaging vectors. Viral supernatants (sups) were harvested from the cells from 24-48 hours post transfection. Viral titres were determined by transducing 3T3 cells, and GFP expression was analysed 48 hours post transduction. All viral transductions were carried out in the presence of 4 mg/ml Polybrene. Suspension cells were spinoculated with virus and Polybrene at 1250g for 90 mins at RT. Cells were re-suspended in appropriate medium for 24 hrs before selection of GFP positive cells by FACS.

### Murine BM transduction and transplantation

C57BL/6 (CD45.2^+^, B6) mice were purchased from Harlan UK or Charles River UK. B6.SJL-*Ptprc*^*a*^/BoyAiTac (CD45.1^+^, B6.SJL) mice bred and housed in the Beatson Biological Service and Research Units. All mouse experiments were approved by United Kingdom (UK) Animal Ethical Committees and performed according to UK Home Office project license 60/4512 (Animal Scientific Procedures Act 1986) guidelines. CD45.2^+^ BM (cKit enriched or sorted for stem and progenitor populations) cells were isolated from 6-8 weeks old mice, and transduced *ex vivo* with control or Trib2 virus in the presence of interleukin (IL)-3 (10 ng/ml), IL-6 (10 ng/ml) and stem cell factor (100 ng/ml). Cells (1×10^6^) were injected intravenously either 24 hours post transduction (HSPCs) or following CFC plating to P3 into lethally irradiated (2×4.25 grays fractionated doses were given three hours apart) or sublethally irradiated (1×4.5 grays) recipients CD45.1^+^. Mice were monitored by periodic tail vein bleedings three weeks after transplantation. Mice were euthanized when they showed clinical symptoms of disease (severe cachexia, lethargy, hunching) and/or elevated WBC counts, and elevating of leukaemic blasts.

### Colony forming cell (CFC) assay

CFC replating assays are frequently used to determine if an oncogene confers self-renewal and proliferative potential to BM stem and progenitor cells *in vitro*. 24hours post-transduction of stem and progenitor populations, 5-20 × 10^4^ transduced cells were resuspended in Methocult GF M3434 (Stem Cell Technologies) methylcellulose media and transferred to 3 × 35 mm culture dishes. Cells were incubated for 7-14 days before colonies were counted, scored and cells analysed by flow cytometry. To assess transformation of stem and progenitor populations, cells were continuously replated to P5. *Trib2* expression in stem and progenitor populations was assessed in a representative set of transductions to confirm effective transgenesis (Supplementary Figure 1D).

### Drug treatments

The cells were treated as indicated with cytarabine (Ara-C), Etoposide (Etop), doxorubicin (DOX), or daunorubicin (DNR) (Sigma-Aldrich), at the concentration indicated. The drugs were stored in DMSO. The BCL2 inhibitor ABT199 was used at the concentrations indicated. The IC50 was experimentally determined as 25.44 μM for PHR expressing cells and 14.07 μM for PHR Trib2 expressing cells.

### Drug synergy studies and data analysis

U937 cells stably transduced with PHR or PHR Trib2 were treated with DNR or/and ABT199 and cell viability was determined using Annexin V and DAPI (Becton Dickinson, Oxford, UK) based flow cytometry analysis to determine live, apoptotic and necrotic cells. Single and combination drug effects were calculated as ‘Fraction effected’ (Fa) of drug treated normalised to untreated cells. CompuSyn software [55] was used to analyse dose dependent effect and evaluate drug synergy by calculation of combination index (CI). CI represents the degree of synergy or antagonism for any two drugs: CI ≥ 1 implies antagonism, CI = 0.9 to 1. additive effect, CI = 0.8 to 0.9 slight synergism, CI = 0.6 to 0.8 moderate synergism, CI = 0.4 to 0.6 synergism, and CI = 0.2 to 0.4 strong synergism.

### Cell lysate preparation

For total cell lysate, cells were lysed using RIPA lysis buffer (50 mM Tris-Hcl pH 7.4, 150 mM Nacl, 1% Triton x-100, 1% Sodium deoxycholate, 0.10% SDS, 1 mM EDTA supplemented with protease and phosphatase inhibtors [1 mM phenylmethylsulfonyl fluoride (PMSF) (Sigma-Aldrich), 2 μg/ml aprotonin (Sigma- Aldrich), 2 μg/ml leupeptin (Sigma-Aldrich), 1 μg/ml pepstatin A (Sigma- Aldrich), 1 mM sodium orthovanadate (Na_3_VO_4_) (Sigma-Aldrich) and 1 mM sodium fluoride (NaF) (Sigma-Aldrich)] followed by incubation on ice for 30 mins. For mitochondrial and cytosolic fractionation, briefly, 5 × 10^6^ cells were washed in PBS and homogenized in a sucrose isolation buffer (10mM Tris–MOPS, 1mM EGTA/Tris, 200mM sucrose, pH 7.4), with a Dounce homogeniser. The homogenised mix was then centrifuged at 600g for 10min at 4°C. Supernatant (containing soluble cytoplasm and heavier membrane organelle) was collected and centrifuged at 7,000g for 10min at 4°C. The supernatant, which constitutes the cytoplasm and other heavy organelles, was collected as a separate fraction for further quantification and analysis. The pellet was separately washed and centrifuged at 7,000g for 10min at 4°C to obtain a pure pellet with mitochondria. It was then lysed using RIPA lysis buffer as above.

### Western blotting

Western blotting was performed using antibodies against Trib2 (1:1000, Santa Cruz B-06, Germany) or in house anti TRIB2 (purified rabiit polyclonal, 1:1000, Patrick Eyers laboratory), BCL2 (1:2000, Cell Signalling), HSP70 (1:5000, Santa Cruz SC-66048), TOMM20 (1:5000 Santa Cruz, SC-17764), β-ACTIN (1:10000 Sigma A5541). Primary antibody detection was by enhanced chemiluminescence (GE Healthcare/Amersham) using horseradish peroxidase−linked secondary antibodies.

### Real time quantitative PCR

Total RNA was extracted from cells using RNeasy® Mini Kit (Qiagen, UK) followed by first strand synthesis to cDNA using High Capacity cDNA Reverse Transcription Kit (Applied Biosystems, UK). Transcript expression of various genes of interest was quantified using Fast SYBR^®^ Green Master Mix (Applied Biosystems), on ABI 7900 quantitative real time PCR machine. Primer sequences described in Supplementary Table 1.

### Mitosis assay

For mitotic index measurement, the cells of interest were fixed using the BD Fixation/Permeabilization solution (BD biosciences) and permeabilised with 90% methanol, followed by incubation with anti-phospho-Histone H3 antibody (pSER10-HH3). DNA staining was performed using DAPI. Flow cytometry analysis was performed using the FACS Canto cytometer (Becton Dickinson) and analysed using FlowJo (Tree Star Incorporation, Oregon, USA).

### Statistics

GraphPad Prism (version 5.03) was used for statistical analysis and graphing. Statistical analyses were performed using the log-rang test and unpaired Student’s *t* test. Statistical significance was determined by a p value indicated as ^*^ (p ≤ 0.05), ^**^ (p ≤ 0.01) and ^***^ (p ≤ 0.001).

### MILE data set analysis

We used the Microarray Innovations in Leukaemia dataset (MILE; GSE13204) [37] to assess the global gene expression of 2096 patients analysed using Affymetrix chips. Expression Console (Thermo-Fisher Scientific) was used for quality control and summarization of the probes’ intensities. The data was normalised using RMA. Gene expression of AML patients was compared with healthy individuals generating a p-value. All those genes presenting a p-value lower than 0.05 and an absolute fold change equal or greater than 2 were considered differentially expressed. To determine if BCL2 is commonly upregulated in AML patients with high expression of TRIB2 (the 25% of the samples with highest expression of TRIB2) the number of samples presenting high levels of expression of either of the genes (the 25% of the samples with highest expression of each gene) were compared. To test if this number was significant the gene expression values were permuted 10,000 times and the number of samples with high expression of both genes compared with the actual value (Monte-Carlo). Not a single permutation reached a higher number of samples than the actual one (p<0.0001). In order to validate this, the median BCL2 expression value of the samples expressing high TRIB2 was calculated (7.05) and using Monte-Carlo again not a single iteration out of 10,000 reached the same expression value (p<0.0001).

## SUPPLEMENTARY MATERIALS FIGURES AND TABLE


