# Supplementary material for: Trib2 expression in granulocyte-monocyte progenitors drives a highly drug resistant acute myeloid leukaemia linked to elevated Bcl2
Source: Oncotarget. 2018 Feb 19;9(19):14977–92. doi: 10.18632/oncotarget.24525 (PMC5871090; doi:10.18632/oncotarget.24525)
Supplement: Supplementary file 1 [file oncotarget-09-14977-s001.pdf]

Trib2 expression in granulocyte-monocyte progenitors drives a highly drug resistant acute myeloid leukaemia linked to elevated Bcl2

SUPPLEMENTARY MATERIALS

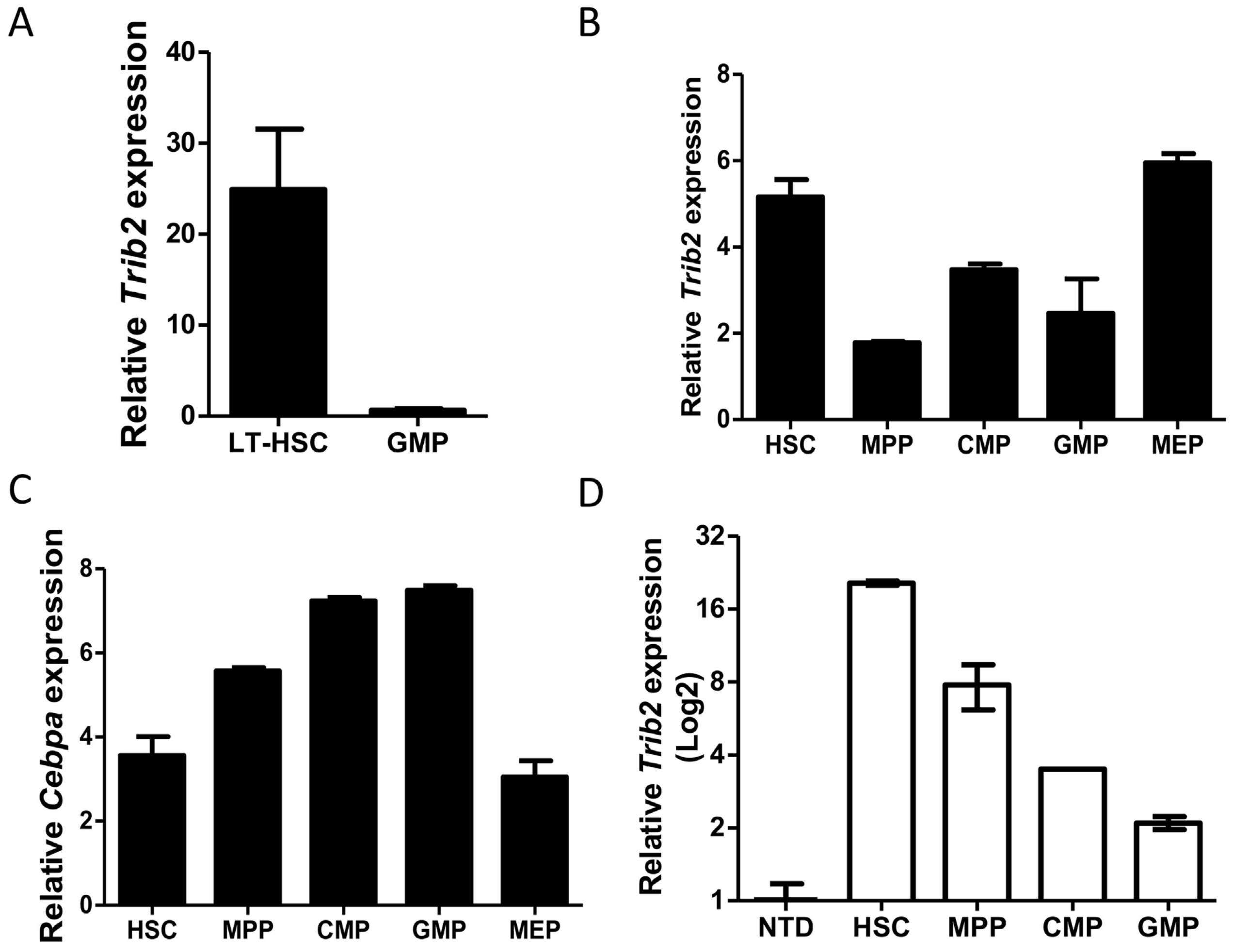

Supplementary Figure 1: *Trib2* expression in stem and progenitor populations. **(A)** Analysis of *Trib2* expression by qPCR in murine LT-HSC and GMP cells isolated from BM by FACS. Bars represent the average of 3 biological replicates each with technical duplicates. Error bars denote +/− SD. Analysis of **(B)** *Trib2* and **(C)** *Cebpa* expression in murine stem and progenitor populations using the RNA-seq dataset (GSE60101). Bars represent the average of n=4 HSC (Lin-, ckit+, Sca1+, Flk2-, CD34+), n=2 MPP (Lin-, ckit+, Sca1+, Flk2+, CD34+), n=2 CMP (Lin-, ckit+, Sca1-, CD34+, FcgRIII int), n=4 GMP (Lin-, ckit+, Sca1-, CD34+, FcgRIII high) and n=4 MEP (Lin-, ckit+, Sca1-, Flk2-, CD34-). Error bars denote +/− SD. **(D)** Analysis of Murine BM cells were sorted for HSC, MPP, CMP and GMP populations, transduced with phrTRIB2 lentivirus and sorted for GFP^{+} cells 24hrs later. Graph represents analysis of *Trib2* expression by qPCR normalised to non-transduced (NTD) control. HPRT1 housekeeping gene was used to normalise the gene expression. Bars represent average of technical triplicates, error bars denote +/− SD.

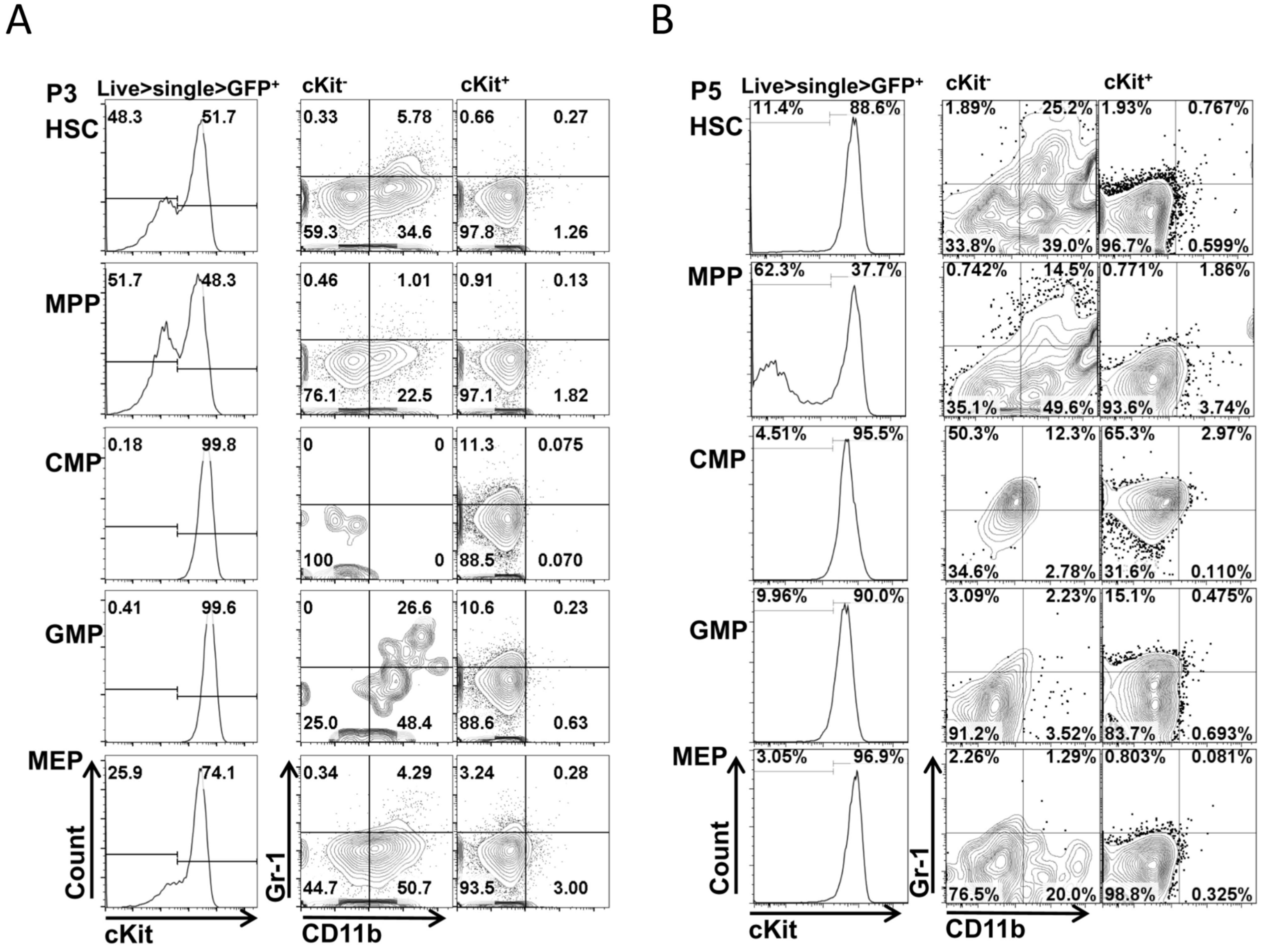

Supplementary Figure 2: (A) Representative flow cytometric analysis of Trib2 transformed P3 stem and progenitor populations showing cKit histograms (left) and CD11b and Gr1 expression through the cKit$^+$ (right) and cKit$^-$ gate (middle). Data are representative of 2 independent experiments. **(B)** Representative flow cytometric analysis of Trib2 transformed P5 stem and progenitor populations showing cKit histograms (left) and CD11b and Gr1 expression through the cKit$^+$ (right) and cKit$^-$ gate (middle). Data are representative of 2 independent experiments.

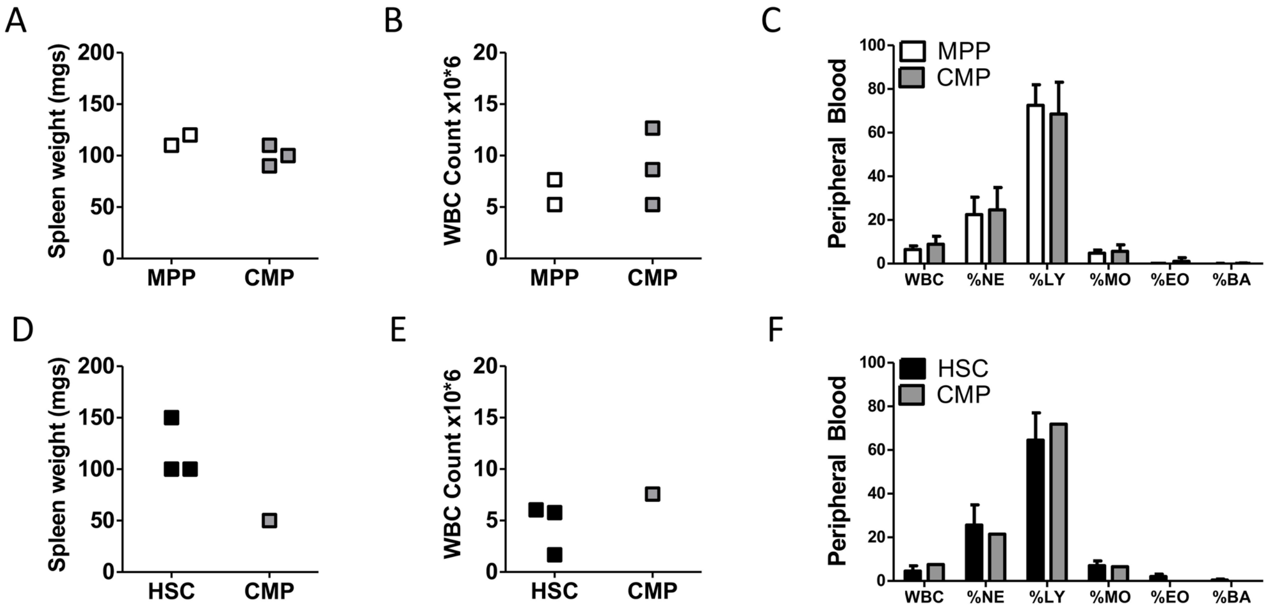

Supplemental Figure 3: Normal myelopoiesis in non-engrafting MPP and CMP transplanted mice, and disease-free HSC and CMP engrafted mice. **(A)** WBC counts and **(B)** spleen weights of MPP and CMP transplanted non-engrafters. Each dot represents an individual mouse. **(C)** The white blood cell differential of the PB was analysed using a HemaVet 950FS. Average percentage neutrophils (NE), lymphocytes (LY), monocytes (MO), eosinophils (EO) and basophils (BA) plotted +/− SD. Average of n=2 MPP, n=3 CMP. HSC and CMP transplanted mice that demonstrated high levels of peripheral engraftment, but did not develop disease, were sacrificed and analysed after 1 year. **(D)** Spleen weights and **(E)** WBC counts of HSC and CMP transplanted engrafters. Each dot represents an individual mouse. **(F)** The white blood cell differential of the PB, average percentage of NE, LY, MO, EO and BA plotted +/− SD. Average of n=3 HSC, n=1 CMP.

Supplementary Table 1: Primers for Real time PCR (SYBR green)

**Human**	**Forward**	**Reverse**
ABL	TGGAGATAACACTCTAAGCATAACTAAAGGT	GATGTAGTTGCTTGGGACCCA
BID3	GGAACCGTTGTTGACCTCAC	GAGGAGCACAGTGCGGAT
BAX	GCTGACATGTTTTCTGACGG	ATGATGGTTCTGATCAGTTCC
Xiap	CGCTCATCGAGGGACGCC	TCCTTATTGATGTCTGCAGGTACAC
Mcl1	CATTCCTGATGCCACCTTCT	TCGTAAGGACAAAACGGGAC
Bcl2	GAGAAATCAAACAGAGGCCG	CTGAGTACCTGAACCGGCA
TRIB2	AGCCAGACTGTTCTACCAGA	GGCGTCTTCCAGGCTTTCCA
Bcl2l11	GGTGAGTCGGATCGCAGC	GGAACGCTTCAACCGCTG
**Mouse**	**Forward**	**Reverse**
HPRT1	GAG AGC GTT GGG CTT ACC TC	ATCGCTAATCACGACGCTGG
Bcl2	CTGAGTACCTGAACCGGCAT	AGAATCCACTCACACCCCAAC
